# Defining a Clinically Meaningful Within-Person Change Threshold for the Psychomotor Vigilance Test in Narcolepsy Type 1

**DOI:** 10.1007/s43441-026-01025-8

**Published:** 2026-08-10

**Authors:** Brian T. Harel, Robert H. Pietrzak, Shruti Dave, Paul Maruff, Heather Romero, Marisa Brimmer, Helen Doll

**Affiliations:** 1 Takeda Development Center Americas, Inc., 500 Kendall Street, Cambridge, MA 02142 USA; 2 Department of Psychiatry, Yale School of Medicine, New Haven, CT USA; 3 Clinical Outcomes Solutions, Tucson, AZ USA; 4 Cogstate Ltd., Melbourne, Australia; 5 Clinical Outcomes Solutions, Folkstone, Kent, UK

**Keywords:** Psychomotor vigilance test, Attention, Narcolepsy type 1, NT1, MWPC, Meaningful within-person change

## Abstract

**Objectives:**

To establish meaningful within-patient change (MWPC) thresholds for the Psychomotor Vigilance Test (PVT) and evaluate whether treatment with the orexin-2 receptor agonist oveporexton (TAK-861) achieved clinically meaningful improvements in attention in people with narcolepsy type 1 (NT1).

**Methods:**

Attention was assessed as number of lapses (reaction times > 500 ms) on the PVT using data from 2 randomized placebo-controlled phase III trials, The First Light (*N* = 166) and The Radiant Light (*N* = 105). Anchor-based methods, supported by distribution-based methods, were used to derive MWPC estimates. Mean changes in square root−transformed PVT lapses (to account for data skewness) were calculated across anchor change categories, with weighted MWPC estimates derived across anchors. Estimates were confirmed with post-hoc receiver operating characteristic (ROC) curve analysis. The proportions of participants meeting MWPC criteria were compared between oveporexton and placebo arms, with odds ratios and 95% confidence intervals.

**Results:**

Across both trials, participants reporting minimal, much, or very much improvement demonstrated progressively fewer PVT lapses. Anchor- and distribution-based analyses supported a reduction of ≥ − 2 PVT lapses, confirmed by ROC curves, as indicating a meaningful improvement in attention. Applying this threshold to unblinded samples, clinically meaningful improvement in attention at day 84 was reported in 66.9% of oveporexton-treated participants versus 25.7% of placebo-treated participants in The First Light and 57.6% versus 17.6% in The Radiant Light (both *P* ≤ 0.0001).

**Conclusions:**

Improvements in attention with oveporexton in people with NT1 were found to be clinically meaningful, with improvement of ≥ − 2 PVT lapses significantly more likely to occur with oveporexton versus placebo.

**Supplementary Information:**

The online version contains supplementary material available at https://doi.org/10.1007/s43441-026-01025-8.

## Introduction

Narcolepsy type 1 (NT1) is defined by the presence of excessive daytime sleepiness and cataplexy [1, 2]; there is, however, growing awareness that adults with NT1 also frequently experience disruptive cognitive symptoms [3–6]. People with NT1 report difficulty in concentrating, focusing, and thinking clearly, which limits their full participation in education and work, reduces their capacity to manage their personal lives, and diminishes their quality of life [5, 7, 8]. Neuropsychological approaches to characterizing cognitive symptoms in NT1 consistently show impairment, most predominantly in attention, as well as executive function and memory, all of which can interfere with daily responsibilities, social participation, and academic or occupational performance [9, 10]. Consequently, interventions designed to reduce symptom burden and improve the lives of adults with NT1 should consider cognitive symptoms.

Phase II and III clinical trials of an orexin-2 receptor agonist, oveporexton (TAK-861), in adults with NT1 included evaluation of the impact of oveporexton on cognitive symptoms associated with NT1 [11–13], including attention, which was measured objectively using the Psychomotor Vigilance Test (PVT), a neuropsychological performance outcome (PerfO) measure widely used in sleep research. The PVT requires individuals to sustain attention over a 10-minute interval to detect visual events that occur randomly, with missed events or slow responses indicating attention lapses. Accordingly, the main PVT endpoint used in clinical trials is the number of lapses made on the test, with greater numbers of lapses indicating a lower ability to sustain attention [14]. Increases in PVT lapses associated with real-world attentional failures have been observed following extended, or off-hour, work shifts in resident physicians [15–18], pilots [19], hospital staff [20], and miners [21], and have been shown to be associated with risky driving behavior [22, 23] and to predict school-related outcomes [24]. PVT lapses have also been used to inform fitness for work examinations in oil rig workers [25] and luggage security screeners [26], and to assess the effects of extended isolation and cognitive changes in astronauts [27–29].

Healthy adults have been shown to have an average of approximately 2 lapses on the 10-minute PVT (upper limit of normal [95th percentile] = 6) [30]. This was shown to be substantially higher in adults with NT1, with mean values of 11.5–17.2 lapses observed in a phase II clinical trial [11]. Oveporexton treatment was shown to significantly reduce the number of PVT lapses made by people with NT1 by approximately 10 lapses over a 12-week period in 2 phase III trials (*P* < 0.0001 versus placebo, both trials) [31]. However, the clinical meaningfulness of this benefit has not been established. The determination of a threshold for meaningful change in PVT lapses in people with NT1 will provide an empirical basis for interpreting the importance of oveporexton-related improvements in attention.

Methods used to determine the meaningful within-patient change (MWPC) of treatment-related benefits include anchor-based and distribution-based approaches, the former using scores from patient-reported outcome (PRO) and clinician-reported outcome (ClinRO) measures to provide an external reference [32]. In anchor-based approaches, the score on the external indicator, termed the anchor, is linked to changes in the outcome of interest (PVT lapses), whereas distribution-based methods are based on statistical evaluation of the outcome’s (PVT lapses) distribution.

The aim of this study was to determine MWPC for PVT lapses in people with NT1, in alignment with recommendations from the US Food and Drug Administration’s (FDA’s) Patient-Focused Drug Development (PFDD) Draft Guidance 4 [33], using blinded data from 2 phase III oveporexton clinical trials, The First Light and The Radiant Light. Derived MWPC values were subsequently evaluated in an unblinded responder analysis, again using trial data, to determine whether treatment with oveporexton resulted in clinically meaningful improvements in attention in people with NT1 in these 2 trials.

## Methods

### Study Design and Participants

The meaningful change analysis used blinded data from 2 randomized, double-blind, placebo-controlled, parallel-group phase III trials of oveporexton (The First Light [TAK-861-3001, NCT06470828, *N* = 166] and The Radiant Light [TAK-861-3002, NCT06505031, *N* = 105]) conducted in adults aged 16–65 years with a confirmed diagnosis of NT1. The First Light randomized participants 2:3:3 to placebo, oveporexton 1 mg twice daily, or 2 mg twice daily, respectively, and The Radiant Light randomized participants 1:2 to placebo or oveporexton 2 mg twice daily. Treatment duration was 12 weeks, with assessments at baseline, day 56, and day 84.

Analyses for the determination of a MWPC threshold for PVT lapses were based on an anchor-based approach, followed by a triangulation step, supported by distribution-based methods and data visualizations, to integrate estimates and produce a range of plausible estimates for the interpretation of change in PVT lapses. Analyses were conducted in accordance with PFDD Draft Guidance 4 and followed the anchor-based triangulation framework [34]. Although not required in formal regulatory frameworks, the identification of MWPCs for outcome measures can also be determined through use of receiver operating characteristic (ROC) analyses [34]. Therefore, to confirm the MWPC threshold for PVT lapses developed in the main analysis, a post-hoc ROC analysis was conducted on the same data to determine the extent to which the PVT lapses threshold values with optimal sensitivity and specificity for the categories from the anchor scales aligned with those from the primary analysis.

### Assessments

Attention was assessed using the PVT, a computerized 10-minute PerfO that objectively quantifies sustained attention. During administration of the PVT, participants respond to visual stimuli appearing at random intervals ranging from 2 to 10 s, and lapses of attention are defined as responses with reaction times > 500 ms [35]. The mean of the 2 daily administrations (morning and afternoon) was used as the endpoint after confirming no systematic error between the 2 administrations. To account for data skewness, a square root transformation of PVT lapses was used in the analyses to assess meaningful change, with back-transformation of the resulting values to the raw scale for purposes of interpretation.

### Anchor Selection

Global (generalized) anchors, and concept-specific anchors, defined from outcome measures closely matching the concept of interest (attention), were identified for consideration. Two PROs were evaluated as potential concept-specific anchor measures: Functional Impacts of Narcolepsy Instrument (FINI; a validated assessment of functional impairment in adults with NT1 [36]) and British Columbia Cognitive Complaints Inventory – Expanded (BC-CCI-E; an expanded version of the original 6-item BC-CCI, a self-reported assessment of cognitive difficulties [37]). Within the FINI, individual Item 9 “Difficulty Concentrating” and FINI – Cognitive Functioning (FINI-CF) domain scores were evaluated. BC-CCI-E total score, as well as 4 individual BC-CCI-E category items (“poor concentration,” “work/school,” “relationships,” and “social, recreational”), were evaluated. Details of each concept-specific anchor measure are provided in the Supplementary Methods.

Patient Global Impression of Severity (PGI-S) and Change (PGI-C) and Clinician Global Impression of Severity (CGI-S) and Change (CGI-C) were used as global anchors to assess patient- and clinician-reported severity or change in overall narcolepsy symptoms over the previous 7 days. These single-item assessments used a 5-point (PGI-S) or 7-point (PGI-C, CGI-S, CGI-C) response scale. Details of each assessment are provided in the Supplementary Methods.

Correlations between each potential anchor variable and square root PVT lapses were calculated to determine the suitability of the anchors to be used to estimate meaningful change, with the threshold being a moderate correlation (≥ 0.30) [32, 38, 39].

### Anchor Categorization for Meaningful Change Analysis

For each selected anchor, participants were classified based on original change (uncollapsed anchor categories) and across broader response categories based on distribution of the change (collapsed anchor categories).

#### FINI-CF

Change scores for FINI-CF Item 9 “Difficulty Concentrating” and FINI-CF total score were calculated as the difference between baseline and post treatment, where negative values represent improvement. Uncollapsed categories on FINI Item 9 ranged from − 4 to + 4, with collapsed categories of − 3 to − 4 (“very much improved”), − 2 (“much improved”), − 1 (“minimally improved”), and 0 to + 4 (“no change/worsening”). Changes in overall patient ratings of their NT1 cognitive symptoms were derived from the following 4 ordered categories of perceived change on FINI-CF total score, based on published MWPCs for this domain derived from normative data from healthy volunteers, with an estimate of − 25 (range, − 20 to − 30) points: “no change or worsening” (FINI-CF total score decrease of < 20), “minimal improvement” (decrease of 20 to < 25 points from baseline), “much improvement” (decrease of 25 to < 30 points from baseline), “very much improvement” (decrease of ≥ 30 points from baseline) [36].

#### BC-CCI-E

Change scores for BC-CCI-E total score, and 4 individual BC-CCI-E category items (“poor concentration,” “work/school,” “relationships,” and “social, recreational”) were calculated as the difference between baseline and post treatment, where negative values represent improvement. Uncollapsed change on each individual item ranged from − 3 to + 3, with collapsed change − 3 (“very much improved”), − 2 (“much improved”), − 1 (“minimally improved”), and 0 to + 3 (“no change/worsened”). Likewise, categorical change on BC-CCI-E was determined from 4 categories of cognitive complaint severity based on total score (0–4, broadly normal; 5–8, mild cognitive complaints; 9–14, moderate cognitive complaints; 15–18, severe cognitive complaints), with participants classified based on how many categories they improved or worsened from baseline: “no change or worsening” (no shift in category or movement to a higher-severity category), “minimal improvement” (improvement by 1 category from baseline), “much improvement” (improvement by 2 categories), and “very much improvement” (improvement by 3 categories).

#### PGI-S and CGI-S

Anchor categories were calculated as the change in the 5-category (PGI-S) or 7-category (CGI-S) anchor score from baseline. For PGI-S, uncollapsed categories ranged from − 4 (“improvement”) to + 4 (“worsening”) and collapsed categories were − 3 to − 4 (“very much improved”), − 2 (“much improved”), − 1 (“minimally improved”), and 0 to + 4 (“no change/worsened”). For CGI-S, uncollapsed categories ranged from − 6 (“improvement”) to + 6 (“worsening”) and collapsed categories were − 3 to − 6 (“very much improved”), − 2 (“much improved”), − 1 (“minimally improved”), and 0 to + 6 (“no change/worsened”).

#### PGI-C and CGI-C

Uncollapsed PGI-C and CGI-C categories were the 7 response categories, ranging from 1 (“very much improved”) to 7 (“very much worse”), with collapsed categories “much/very much improved” (1–2), “minimally improved” (3), “no change” (4), “minimally worse” (5), and “much/very much worse” (6–7).

### Anchor-Based Analyses

Anchor-based methods applied at day 84 were used to link change in square root PVT lapses to clinician-reported changes on the CGI-S and CGI-C and patient-reported changes on the PGI-S, PGI-C, and FINI-CF and BC-CCI-E scores. These analyses included the calculation of mean change in square root PVT lapses across ordered categories of anchor change, with the magnitude of PVT lapse reduction corresponding to each anchor change category evaluated to identify the smallest reliable performance improvement consistent with a patient-perceived benefit. Within-patient differences were examined for statistical significance (*P* < 0.05), clinical magnitude (Cohen’s d ≥ 0.50), and nonoverlapping 95% confidence intervals (CIs) compared with the “no change” group, to determine the extent to which any differences observed reflected statistically and clinically meaningful, rather than random, variation.

Post-hoc ROC analysis was conducted to identify best cut-points (BCPs) for 1 +, 2 +, or 3 + category changes for each anchor. An average BCP for each anchor category change was calculated from the 3 square root PVT lapse values that: (1) had the smallest difference on the ROC curve from the 0,1 point in the ROC space; (2) maximized the value of sensitivity minus specificity; and (3) maximized the Youden’s Index [40]. Given that BCPs ‘cut’ between categories, BCPs would be expected to be slightly smaller than mean change values [34].

### Empirical Cumulative Distribution Function (eCDF) and Probability Density Function (PDF)

To visualize separation of change distributions across anchor categories, eCDF and PDF curves were plotted for change in square root PVT lapses from baseline to day 84. These graphical approaches provided complementary evidence regarding magnitude and consistency of change in relation to patient-reported improvement on the anchors reflecting their cognitive symptoms. Distinct and nonoverlapping distributions were interpreted as supporting differentiation of PVT lapses across anchor categories.

### Triangulation of Anchor-Based Estimates

To provide a reliable and transparent approach to triangulation [41], clinically meaningful change was defined as the smallest reliable improvement in square root PVT lapses associated with participant- and clinician-perceived improvement, determined by 4 converging criteria: minimal overlap of 95% CIs with the “no change” group, a moderate-to-large effect size (Cohen’s d > 0.50), statistically significant within-group improvement (*P* < 0.05), and within-change groups having ≥ 10 participants. Weighted estimates of meaningful change were then derived across anchor categories, weighting by the anchor correlation or by the inverse variance [41]. Weighted estimates were produced both across all anchors and across concept-specific anchors only.

### Distribution-Based Analyses

Distribution-based methods were used to provide statistical support for the MWPC threshold. Indices included the baseline standard deviation (SD), half SD (0.5 SD), and standard error of measurement (SEM), calculated as *SEM = SD × √(1 – ICC)*, where the intraclass correlation coefficient (ICC[A,1]) reflected test-retest reliability between morning and afternoon PVT administrations at baseline. The anchor-based estimates being larger than the 0.5 SD and SEM values was interpreted as evidence that the proposed MWPC exceeded measurement error.

### Final Estimates of Meaningful Change

Results from anchor-based, eCDF/PDF, and distribution-based analyses were evaluated together to derive a final MWPC estimate and range for PVT lapses. This estimate prioritized the anchor-based findings with support from distributional data to ensure a balance between statistical robustness and clinical relevance. The estimate and range were back-transformed to the scale of raw number of PVT lapses for interpretability.

### Responder Analyses

The derived MWPC threshold was applied to unblinded clinical trial data to estimate the proportion of participants in each treatment arm who achieved a clinically meaningful improvement in attention at completion of the trial. This provided a practical measure of real-world relevance of the effect of oveporexton on attention.

All analyses were conducted using SAS version 9.4 (SAS Institute, Cary, NC, USA).

## Results

### Sample Characteristics

Across both phase III trials, the mean participant age was 31.2 (SD, 11.22; range, 16–65) years. Slightly over half of the participants were female (54.2%). Across both the placebo and treatment groups, mean PVT lapses were 15.19 (SD, 14.88) at baseline, compared with 10.36 (SD, 13.33) at day 84. Mean square root PVT lapses were 3.43 (SD, 1.86) at baseline and 2.65 (SD, 1.83) at day 84. Complete sample characteristics are shown in Tables 1 and 2.

**Table 1:** Participant characteristics at baseline

Parameter	Statistic	The First Light (*N* = 166)	The Radiant Light (*N* = 105)	PVT-pooled (*N* = 271)
Age (years)	*n*	166	105	271
	Mean (SD)	31.5 (11.32)	30.7 (11.10)	31.2 (11.22)
	Median	28.5	27.0	28.0
	Q1, Q3	22.0, 39.0	22.0, 37.0	22.0, 39.0
	Min, max	16, 65	18, 62	16, 65
	*n* missing	0	0	0
Sex, *n* (%)	Male	69 (41.6)	55 (52.4)	124 (45.8)
	Female	97 (58.4)	50 (47.6)	147 (54.2)
	*n* missing	0	0	0
Race, *n* (%)	White	58 (34.9)	47 (44.8)	105 (38.7)
	Black or African American	6 (3.6)	0	6 (2.2)
	Asian	26 (15.7)	21 (20.0)	47 (17.3)
	American Indian or Alaska Native	1 (0.6)	0	1 (0.4)
	Multiple	1 (0.6)	0	1 (0.4)
	Unknown	59 (35.5)	37 (35.2)	96 (35.4)
	Not reported	15 (9.0)	0	15 (5.5)
	*n* missing	0	0	0
Ethnicity, *n* (%)	Hispanic or Latino	13 (7.8)	0	13 (5.6)
	Not Hispanic or Latino	74 (44.6)	68 (100.0)	142 (60.7)
	Not reported	17 (10.2)	0	17 (7.3)
	Unknown	62 (37.3)	0	62 (26.5)
	*n* missing	0	37	37

*n* = number of participants with nonmissing data used as the denominator.

Max, maximum; min, minimum; PVT-pooled, pooled patient-reported outcome population; Q1, 25th percentile; Q3, 75th percentile; SD, standard deviation.

**Table 2:** Clinical outcome assessment score distribution at baseline and day 84 (PVT-pooled, *N* = 271)

Clinical measure	Statistic	Baseline	Day 84
PVT lapses	*n*	271	256
	Mean (SD)	15.19 (14.880)	10.36 (13.327)
	Median	10.00	6.00
	Q1, Q3	4.00, 22.00	2.00, 12.25
	Min, max	0.00, 76.50	0.00, 76.00
	*n* missing	0	1
Square root PVT lapses	*n*	271	256
	Mean (SD)	3.43 (1.856)	2.65 (1.830)
	Median	3.16	2.45
	Q1, Q3	2.00, 4.69	1.41, 3.50
	Min, max	0.00, 8.75	0.00, 8.72
	*n* missing	0	1
FINI-CF	*n*	271	257
	Mean (SD)	51.24 (25.964)	25.21 (26.447)
	Median	50.00	20.00
	Q1, Q3	30.00, 70.00	5.00, 40.00
	Min, max	0.00, 100.00	0.00, 100.00
	*n* missing	0	0
BC-CCI-E total score	*n*	271	257
	Mean (SD)	8.32 (4.441)	4.81 (4.442)
	Median	8.00	4.00
	Q1, Q3	5.00, 12.00	1.00, 7.00
	Min, max	0.00, 18.00	0.00, 18.00
	*n* missing	0	0

BC-CCI-E, British Columbia Cognitive Complaints Inventory – Extended; FINI-CF, Functional Impacts of Narcolepsy Instrument – Cognitive Functioning; max, maximum; min, minimum; PVT, Psychomotor Vigilance Test; PVT-pooled, pooled patient-reported outcome population; Q1, 25th quartile; Q3, 75th quartile; SD, standard deviation.

### Anchor-Based Meaningful Change Estimation

Change from baseline in square root PVT lapses correlated moderately (≥ 0.30) with change in all anchor scores at either day 56 or 84 in ≥ 1 of the 2 trials, confirming their suitability as anchors (Appendix Table 1 in Supplementary Materials). Of the potential concept-specific anchors assessed (FINI-CF, FINI Item 9, BC-CCI-E total score, and BC-CCI-E category items “poor concentration,” “work/school,” “relationships,” and “social, recreational”), all anchors were retained for derivation of meaningful change in The First Light, whereas in The Radiant Light, only FINI-CF, FINI Item 9, BC-CCI-E total score, and BC-CCI-E Item 2 were retained. The PGI-S, CGI-S, PGI-C, and CGI-C global anchors were used in both studies.

For each trial, reduction in square root PVT lapses demonstrated a clear, graded association with patients’ ratings of improvement in their cognitive/overall symptoms determined from all anchor measures; data for FINI-CF, FINI Item 9, BC-CCI-E total score, BC-CCI-E category items (“poor concentration,” “work/school,” “relationships,” and “social, recreational”), PGI-S, CGI-S, PGI-C, and CGI-C are shown in Table 3. In both trials, participants who reported “no change or worsening” generally showed negligible change in square root PVT lapses, whereas those reporting “minimal”/1-category, “much”/2-category, or “very much”/≥ 3-category improvement demonstrated progressively greater reductions in square root PVT lapses, indicating greater improvement on the PVT. Overall, improvement was slightly greater in The First Light, with effect sizes for those reporting minimal or greater improvement ranging from 0.21 to 1.44 and 0.15 to 0.98 in The Radiant Light (Table 3).

When examining meaningful change for all anchors and at each study time point, the approach taken was to identify the group with the smallest change in square root PVT lapses, which could be considered meaningful to participants, and which could reliably distinguish participants indicating change on the respective anchor from those who indicated no change. Identified groups used to indicate meaningful change are shown in bold in Table 3.

**Table 3:** Change in square root PVT lapses (with square root values back-transformed to raw values) by patient-reported cognitive improvement category (FINI-CF, FINI-CF Item 9, and BC-CCI-E Total and Items 2, 7, 8, and 9) and global change on the PGI-S, PGI-C, CGI-S, and CGI-C at day 84

Measure anchor category	The First Light				The Radiant Light			
	Δ SQRT PVT lapses, mean (SD)	Δ Raw PVT lapses, mean (95% CI)	*n*	Effect size (d)	Δ SQRT PVT lapses, mean (SD)	Δ PVT lapses, mean (95% CI)	*n*	Effect size (d)
FINI-CF score								
No change/worsening	–0.1 (1.46)	–0.01 (–0.22, 0.07)	61	0.050	–0.2 (1.58)	–0.04 (–0.50, 0.06)	45	0.143
** 20- to < 25-point improvement**	–1.5 (1.52)*	**–2.25 (–7.62**,** − 0.04)**	**8**	**0.743**	**–0.5 (1.43)**	**–0.25 (–7.51**,** 3.31)**	**4**	**N/A**
25- to < 30-point improvement	–0.8 (1.56)	–0.64 (–2.86, 0.04)	13	0.377	–0.2 (2.09)	–0.04 (–4.71, 2.86)	7	0.147
≥ 30-point improvement	–1.7 (1.80)***	–2.89 (–4.54, − 1.66)	74	0.858	–0.8 (1.54)**	–0.64 (–1.56, − 0.10)	44	0.482
FINI-CF Item 9: Difficulty Concentrating								
No change/worsening	–0.1 (1.43)	–0.01 (–0.22, 0.13)	49	0.028	0.1 (1.27)	0.01 (–0.14, 0.25)	35	0.039
** 1-category improvement**	–1.3 (1.50)***	**–1.69 (–3.28**,** − 0.58)**	**34**	**0.646**	**–0.8 (1.95)**	**–0.64 (–2.96**,** 0.03)**	**19**	**0.484**
2-category improvement	–1.3 (1.95)***	–1.69 (–3.46, − 0.48)	45	0.642	–0.7 (1.33)*	–0.49 (–1.54, − 0.01)	24	0.417
3-category improvement	–1.8 (1.86)***	–3.24 (–6.30, − 1.14)	28	0.898	–0.9 (1.84)*	–0.81 (–2.86, − 0.004)	22	0.544
BC-CCI-E total score								
No change/worsening	–0.6 (1.73)**	–0.36 (–1.00, − 0.04)	72	0.301	0.1 (1.33)	–0.16 (–0.53, − 0.001)	55	0.049
** 1-category improvement**	−1.1 (1.78)***	**–1.21 (–2.43**,** − 0.37)**	**57**	**0.546**	–1.1 (1.80)**	**–0.81 (–2.28**,** − 0.07)**	**28**	**0.673**
2-category improvement	−1.6 (1.80)***	–2.56 (–5.62, − 0.61)	22	0.791	–1.2 (1.33)**	–2.56 (–5.76, − 0.59)	15	0.751
≥ 3-category improvement	−2.9 (1.13)**	–8.41 (–18.23, − 2.13)	5	1.438	–1.9 (2.13)	–2.25 (–143, 79.2)	2	N/A
BC-CCI-E Item 2: Poor Concentration								
No change/worsening	–0.5 (1.58)*	–0.25 (–0.77, − 0.001)	56	0.228	0.1 (1.42)	0.01 (–0.11, 0.27)	46	0.059
** 1-category improvement**	–1.1 (1.96)***	**–1.21 (–2.34**,** − 0.35)**	**69**	**0.535**	–0.7 (1.66)*	–0.49 (–1.69, − 0.01)	32	0.435
2-category improvement	–1.6 (1.04)***	–2.56 (–4.04, − 1.25)	23	0.787	–1.6 (1.44)***	**–2.56 (–5.62**,** − 0.61)**	**15**	**0.978**
3-category improvement	–2.4 (2.38)*	–5.76 (–19.18, − 0.17)	8	1.204	–0.9 (1.39)	–0.81 (–4.75, 0.14)	7	0.557
BC-CCI-E Item 7: Difficult for Me to Do My Job								
No change/worsening	–0.6 (1.69)**	–0.36 (–1.12, − 0.04)	62	0.319	–	–	–	–
1-category improvement	–0.7 (1.83)**	–0.49 (–1.56, − 0.04)	49	0.366	–	–	–	–
** 2-category improvement**	–1.8 (1.81)***	**–3.24 (–6.40**,** − 1.14)**	**26**	**0.906**	–	–	–	–
3-category improvement	–1.7 (1.60)***	–2.89 (–6.05, − 0.85)	19	0.848	–	–	–	–
BC-CCI-E Item 8: Difficult to Have a Good Relationship								
No change/worsening	–0.7 (1.88)**	–0.49 (–1.19, − 0.07)	81	0.340	–	–	–	–
1-category improvement	–0.9 (1.67)***	–0.81 (–1.96, − 0.20)	50	0.463	–	–	–	–
** 2-category improvement**	–1.9 (1.27)***	**–3.61 (–6.25**,** − 1.82)**	**21**	**0.967**	–	–	–	–
3-category improvement	–3.2 (0.54)**	–10.24 (–16.40, − 5.43)	4	N/A	–	–	–	–
BC-CCI-E Item 9: Difficult to Enjoy Social Activity								
No change/worsening	–0.5 (1.86)*	–0.25 (–1.04, − 0.001)	56	0.264	–	–	–	–
1-category improvement	–0.8 (1.57)***	–0.64 (–1.56, − 0.18)	57	0.418	–	–	–	–
** 2-category improvement**	–1.6 (1.86)***	**–2.56 (–5.24**,** − 0.94)**	**33**	**0.820**	–	–	–	–
3-category improvement	–2.3 (1.34)***	–5.29 (–10.82, − 1.88)	10	1.172	–	–	–	–
PGI-S								
No change/worsening	–0.3 (1.71)	–0.09 (–0.79, 0.05)	38	0.165	0.3 (1.67)	0.09 (–0.10, 1.00)	27	0.211
** 1-category improvement**	–1.0 (1.86)***	**–1.0 (–2.13**,** − 0.20)**	**54**	**0.478**	–0.4 (1.32)	–0.16 (–0.85, 0.01)	28	0.253
2-category improvement	–1.2 (1.50)***	–1.44 (–2.82, − 0.58)	43	0.614	–1.0 (1.62)**	**–1.0 (–2.56**,** − 0.15)**	**30**	**0.616**
≥ 3-category improvement	–1.8 (2.03)***	–3.24 (–7.40, − 0.76)	21	0.901	–1.1 (1.35)**	–1.21 (–3.31, − 0.10)	15	0.663
PGI-C								
Much/very much worse	–0.6 (1.83)	–0.36 (–3.76, 0.46)	10	0.317	0.6 (0.92)	0.36 (–0.01, 1.66)	9	0.366
Minimally worse	2.1 (0.81)*	4.41 (0.01, 16.56)	3	N/A	0.1 (1.58)	0.01 (–14.9, 15.8)	3	N/A
No change	–0.2 (1.43)	–0.04 (–1.25, 0.66)	11	0.079	0.6 (0.99)*	0.36 (0.003, 1.21)	16	0.357
Minimally improved	–0.6 (2.19)	–0.36 (–2.59, 0.19)	20	0.293	–0.8 (1.67)	–0.64 (–5.34, 0.59)	7	0.480
** Much/very much improved**	–1.3 (1.67)***	**–1.69 (–2.46**,** − 0.88)**	**112**	**0.630**	–0.9 (1.63)***	**–0.81 (–1.64**,** − 0.23)**	**65**	**0.547**
CGI-S								
No change/worsening	–0.2 (1.88)	–0.04 (–0.94, 0.42)	23	0.082	0.6 (1.06)**	0.36 (0.02, 0.98)	27	0.356
1-category improvement	–0.4 (1.69)	–0.16 (–1.14, 0.05)	29	0.214	–0.2 (1.72)	–0.04 (–1.17, 0.40)	18	0.141
** 2-category improvement**	–1.1 (1.55)***	**–1.21 (–2.62**,** − 0.29)**	**34**	**0.540**	–1.5 (1.87)*	**–2.25 (–6.97**,** − 0.14)**	**13**	**0.938**
≥ 3-category improvement	–1.4 (1.69)***	–1.96 (–3.42, − 1.06)	67	0.725	–1.0 (1.32)***	–1.00 (–2.02, − 0.35)	41	0.624
CGI-C								
Much/very much worse	0.2 (2.57)	0.04 (–6.30, 8.29)	6	0.091	0.2 (0.62)	0.04 (–0.64, 1.39)	4	N/A
Minimally worse	0.8 (0.67)	0.64 (–0.79, 5.86)	3	N/A	0.7 (0.79)	0.49 (–0.07, 2.96)	5	0.452
No change	–0.2 (1.32)	–0.04 (–0.90, 0.26)	15	0.110	0.5 (1.07)*	0.25 (0.002, 0.94)	23	0.315
Minimally improved	–0.8 (1.97)*	–0.64 (–2.25, − 0.01)	32	0.396	–1.5 (1.92)	–2.25 (–12.4, 0.26)	6	0.934
** Much/very much improved**	–1.3 (1.65)***	**–1.69 (–2.76**,** − 1.00)**	**99**	**0.669**	–0.9 (1.59)***	**–0.81 (–1.77**,** − 0.27)**	**61**	**0.575**

Target change groups are indicated in bold. Statistically significant change: *P < 0.05; **P < 0.01; ***P < 0.001. N/A = not applicable because *n* < 5 participants in group.

BC-CCI-E, British Columbia Cognitive Complaints Inventory – Extended; CI, confidence interval; CGI-C, Clinician Global Impression of Change; CGI-S, Clinician Global Impression of Severity; FINI-CF, Functional Impacts of Narcolepsy Instrument – Cognitive Functioning; N/A, not applicable; PGI-C, Patient Global Impression of Change; PGI-S, Patient Global Impression of Severity; PVT, Psychomotor Vigilance Test; SD, standard deviation; SQRT, square root.

### Interpretation of eCDF and PDF Curves

**Fig. 1:**
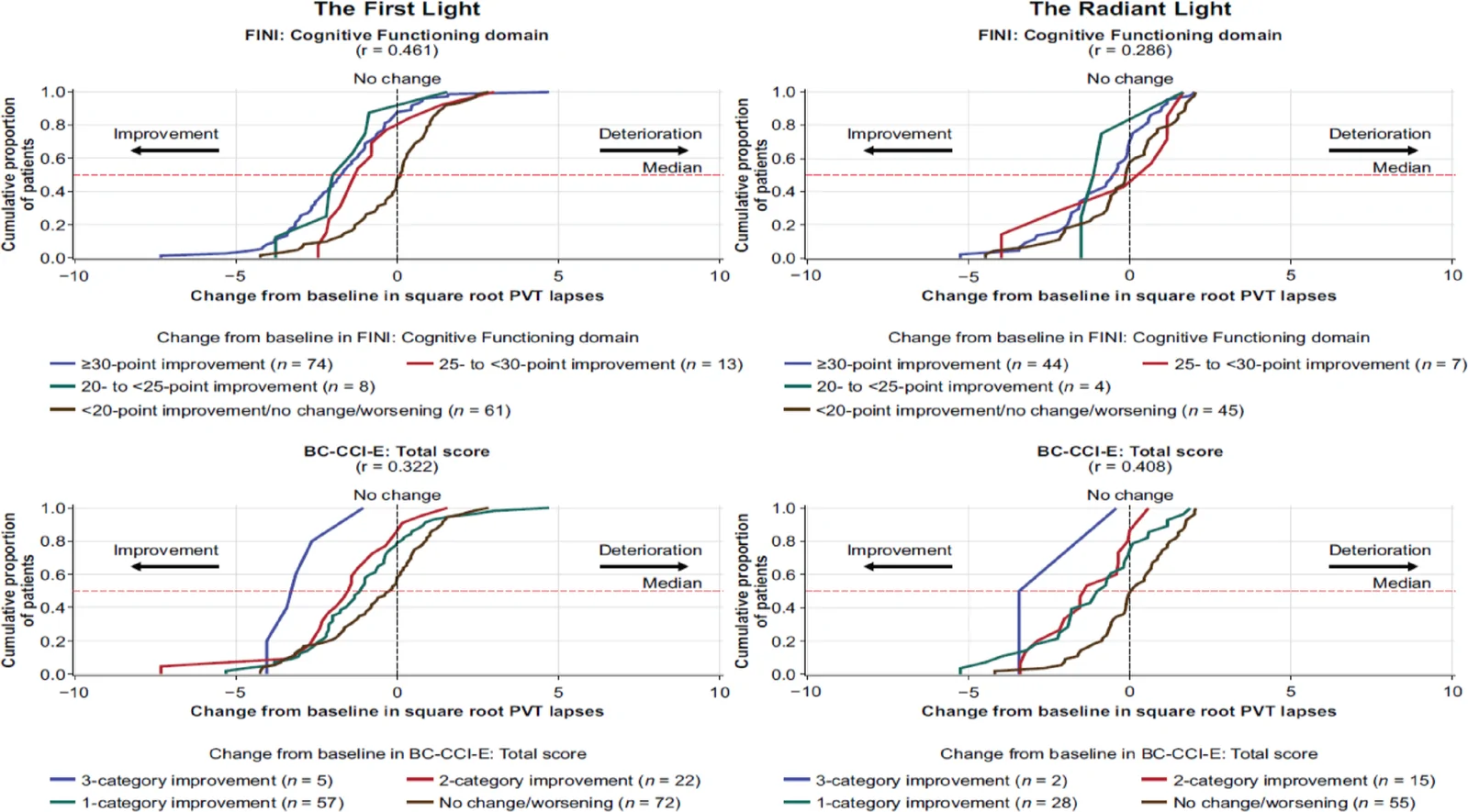
eCDF curves of the change in square root PVT lapses from baseline to day 84 for the FINI-CF domain and BC-CCI-E total score anchors in The First Light and The Radiant Light. BC-CCI-E, British Columbia Cognitive Complaints Inventory – Extended; eCDF, empirical cumulative distribution function; FINI-CF, Functional Impacts of Narcolepsy Instrument – Cognitive Functioning; PVT, Psychomotor Vigilance Test

**Fig. 2:**
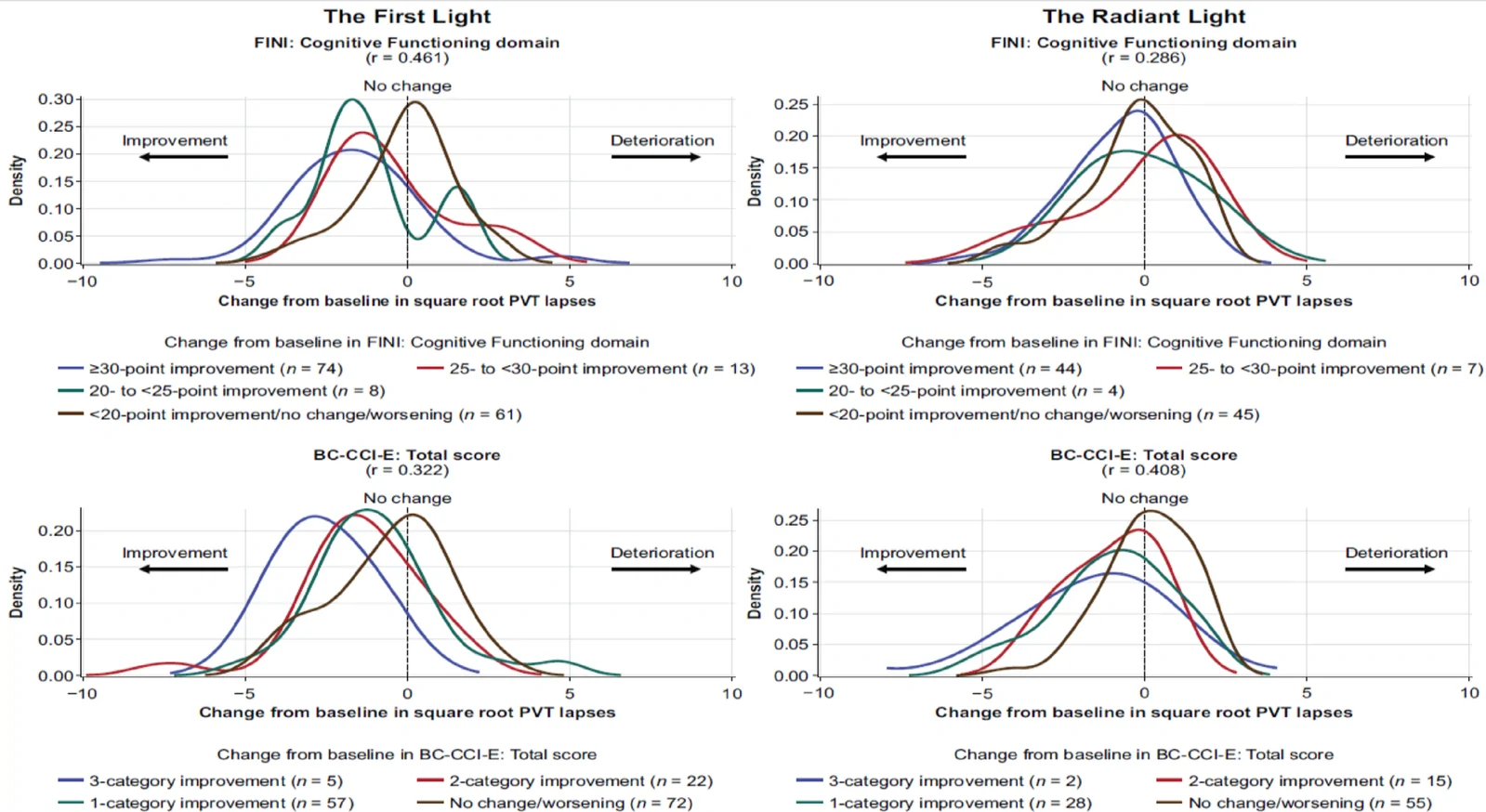
PDF curves of the change in square root PVT lapses from baseline to day 84 for the FINI-CF domain and BC-CCI-E total score anchors in The First Light and The Radiant Light. BC-CCI-E, British Columbia Cognitive Complaints Inventory – Extended; FINI-CF, Functional Impacts of Narcolepsy Instrument – Cognitive Functioning; PDF, probability density function; PVT, Psychomotor Vigilance Test

eCDF and PDF curves were used to provide complementary visual evidence to support validity of the anchor-based MWPC estimate. Example figures are shown for the FINI-CF domain and BC-CCI-E in Fig. 1 (eCDF curves) and Fig. 2 (PDF curves); eCDF and PDF curves for PGI-S, PGI-C, CGI-S, and CGI-C are shown in Appendix Figs. 1, 2, 3 and 4 in Supplementary Materials. Across all anchors, eCDF curves demonstrated clear monotonic separation of distribution of change in PVT lapse scores across ordered categories of patient-reported improvement, with greater shifts observed for participants reporting “much” or “very much” improvement. Corresponding PDF plots revealed distinct, nonoverlapping peaks between improvement categories, indicating that greater increases in anchor scores were consistently associated with greater reductions in PVT lapses.

### Triangulation of Anchor-Based Estimates

Mean change in square root PVT lapses in the target anchor category ranged, across all anchors, from −1.0 to −1.9 in The First Light and from −0.8 to −1.6 in The Radiant Light (Table 3). Overall (inverse variance and correlation) weighted mean square root PVT lapse values in the target anchor categories were −1.4 (−1.38 and −1.41) in The First Light and −1.2 (−1.15 and −1.21) in The Radiant Light. Estimates from the concept-specific anchors were generally larger, particularly in The First Light, than estimates that also included the global anchors, but rounded values were the same.

### Distribution-Based Estimates

Distribution-based estimates of MWPC for PVT lapses were consistent across trials (Table 4). In The First Light, for square root PVT lapses, the 0.5 SD was 1.00 and SEM was 0.96; in The Radiant Light, the corresponding values were 0.81 and 0.84, respectively. On back-transforming these values to raw PVT lapses, in The First Light, the 0.5 SD was 0.99 and SEM was 1.92; in The Radiant Light, the values were 0.65 and 1.35 (Table 4). These estimates indicate that the MWPC threshold for square root PVT lapses should be as large as or greater than 0.84 (The Radiant Light) to 0.96 (The First Light) for square root PVT lapses to maximize the probability that any clinically significant improvement in lapses is also statistically meaningful.

**Table 4:** Distribution-based estimates of meaningful change in PVT lapses (square root values back-transformed to raw values)

Study	*N*	SQRT PVT lapses					PVT lapses			
		Baseline SD	0.2 SD	0.5 SD	ICC(A,1)	SEM*	Baseline SD	0.2 SD	0.5 SD	SEM*
The First Light	166	1.99	0.398	0.995	0.765	0.965	3.96	0.158	0.990	1.920
The Radiant Light	105	1.61	0.322	0.805	0.730	0.837	2.59	0.104	0.648	1.347

*SEM = baseline SD × SQRT (1 – ICC).

ICC(A,1), intraclass correlation coefficient; PVT, Psychomotor Vigilance Test; SD, standard deviation; SEM, standard error of the mean; SQRT, square root.

### Final Meaningful Change Threshold

Based on analysis of anchor-based and distribution-based findings and visual plots, a single-point estimate of −1.3, with a range from −1.0 to −1.9, was selected to represent meaningful change in square root PVT lapses (i.e., smallest reliable change that was distinguishable from “no change/worsening”). These estimates were larger than the distribution-based SEM estimates of 0.84–0.96. Back-transformation of these results with conservative rounding-up led to the MWPC threshold being defined as an improvement of −2 PVT lapses (raw score), with a range of improvement of −1 to −4 lapses.

### PVT Change at Day 84 by Degree of Anchor Change

The MWPC estimate of −2, with a range of −1 to −4, was identified as the smallest reliable performance improvement associated with a patient-perceived benefit per EU and US regulatory guidelines [33, 42]. This value of −2 corresponds to a “moderate improvement” (largest upper bound of the CI [UCI] of the minimal improvement group −1.32, back-transformed number of lapses −1.74, rounded to −2) on the anchors. Change estimates were also identified for “much improvement” (range of “much/very much improvement” group means −1.57 to −1.84, back-transformed to −2.46 to −3.39, rounded to −3 to −4). Finally, change estimates of −5 were identified for “very much improvement” (largest UCI of “much/very much improvement” group −2.19, back-transformed to −4.80, rounded to −5).

#### Post-Hoc ROC Analyses

Post-hoc ROC BCPs and illustrative ROC curves are provided in Appendix Tables 2 and 3 and in Appendix Figs. 5 and 6 in Supplementary Materials. The ROC BCPs, while also showing monotonic trends, were generally smaller than the respective mean anchor category values (Appendix Tables 2 and 3 in Supplementary Materials). Thus, the lower bound value of −1 in the identified MWPC range of −1 to −4 corresponded to a “minimal improvement”, as identified by the ROC BCPs for square root PVT lapses for the 1+ (minimal improvement) group, which, for the concept-specific anchors in The First Light, ranged from −0.40 to −1.27 (average −0.75, back transformed to −0.56, rounded to −1). Likewise, the lower bound value of −3 of the range − 3 to −4 indicating “much improvement” was consistent with the ROC BCPs for 3+ (much improvement) for the concept-specific anchors in The First Light (average −1.71, back-transformed to −2.92, rounded to −3) (Appendix Table 2 in Supplementary Materials). Finally, the estimate of −5 for “very much improvement” was consistent with the 5 largest concept-specific ROC BCPs for 3+ (much improvement) in The First Light which ranged from −1.10 to −2.58, average − 2.04, back-transformed to −4.16, rounded to −5 (Appendix Table 2 in Supplementary Materials).

### Responder Analysis

Applying the threshold for clinically meaningful change in PVT lapses (−2 PVT lapses) to the unblinded trial data (i.e., separated by treatment group) identified that this threshold was significantly more likely to be achieved in the groups receiving oveporexton. In The First Light and The Radiant Light trials, meaningful improvement occurred in 66.9% and 57.6% of oveporexton-treated participants and 25.7% and 17.6% of placebo-treated participants, respectively, at day 84, and was statistically significantly superior to placebo (*P* ≤ 0.0001; OR, 5.85; 95% CI, 2.51–13.65; and OR, 6.33; 95% CI, 2.31–17.35). Thus, in both trials, the odds of clinically meaningful improvement in PVT lapses were 6 times larger following treatment with oveporexton than with placebo. When applying the larger threshold of −5 PVT lapses, which was associated with the greatest patient-perceived benefit (“very much improvement”), meaningful improvement occurred in 50.4% and 39.4% of the treated groups versus 17.1% and 8.8% of the placebo groups, respectively, with improvement in the treatment groups at day 84 across both studies being statistically significantly superior to placebo (OR, 4.914; 95% CI, 1.903–12.687; and OR, 6.717; 95% CI, 1.86–24.25, respectively).

## Discussion

To our knowledge, this analysis is the first to derive MWPC threshold values for a neuropsychologically defined cognitive PerfO that is in accordance with the best practice framework outlined in FDA PFDD Draft Guidance 4 [33], and with the triangulation approach proposed by Meunier et al. [34], and provides quantitative evidence for the definition of MWPC in attention, measured using the PVT, in adults with NT1 treated with oveporexton, a selective orexin-2 receptor agonist. Using both concept-specific and global anchors, an improvement of −2 PVT lapses (range, −1 to −4) was identified as the threshold for meaningful improvement on the basis that it corresponded to meaningful patient reports of reduced cognitive and overall narcolepsy symptoms following treatment. These estimates were larger than lower bound estimates derived by distribution-based methods to exclude measurement error [35, 43].

The graded correspondence between drug-related improvement on an objective assessment of attention, assessed using a neuropsychological PerfO, and drug-related improvement in patients’ ratings of their own cognitive difficulties, assessed using PROs, is consistent with neurobiological models that link restoration of orexin neurotransmission to enhanced cortical arousal, vigilance, and executive attention [44]. Prior psychometric research has shown that lapses of attention measured using the PVT are among the most sensitive and reliable indices of attention impairment in narcolepsy, with excellent test-retest reliability (ICC, 0.82–0.89) and internal consistency (Cronbach’s α, 0.81–0.86), and sensitivity to detect change [43].

The current analysis, by examining the relationship between cognitive difficulties and the PVT and showing that individuals’ ratings of their cognitive difficulties corresponded to meaningful changes in the PVT, provided further evidence of the validity of the PVT. Participants with NT1 enrolled in The First Light and The Radiant Light phase III oveporexton clinical trials who indicated reduced subjective cognitive difficulties in response to treatment also showed a reduction in attentional lapses on the PVT [31], with the magnitude of this improvement in attention increasing progressively with the magnitude of reduction in cognitive difficulties. The post-hoc ROC results were consistent with those from the main analysis. These findings converge to support the PVT as a fit-for-purpose PerfO for assessing attention in NT1 clinical trials.

The MWPC for PVT lapses, developed from the phase III trials using blinded analyses (i.e., agnostic to treatment group assignment), was applied to unblinded trial data to explore the clinical significance of the observed group-level treatment effect. Using an improvement of ≥ −2 lapses as the criteria, clinically meaningful improvement was identified in 57.6–66.9% of oveporexton-treated participants and in only 17.6–25.7% of participants receiving placebo. Even when using a threshold of an improvement in PVT performance of −5 lapses, which aligned with patients’ ratings of their cognitive symptoms being “very much improved” (highest category available on the PROs), clinically meaningful improvement in attention was approximately 5 times more frequent in each oveporexton treatment group than in the placebo group (39.4–50.4% for oveporexton-treated participants versus 8.8–17.1% for participants receiving placebo). Taken together, results indicate that oveporexton treatment was associated with a clinically meaningful improvement in attention as measured by a meaningful decrease in PVT lapses.

This also accords with observations of the effect of oveporexton on attention in adults with NT1 [11, 31], with group differences being statistically significant and of a large magnitude. These findings are also consistent with the outcome of a meta-analysis of data for PVT lapses from the application of the test in clinical trials [14].

The findings from this analysis have important implications for clinical, research, and regulatory evaluation. First, the −2 lapse PVT threshold provides an empirically grounded benchmark for interpreting the extent to which any improvement in attention observed in adults with NT1 participating in clinical trials investigating drug effects on cognitive symptoms can be classified as clinically meaningful. Second, integrating objective performance measures with patient-reported measures exemplifies a patient-focused approach to clinical outcome assessment, consistent with US and EU regulatory guidance for defining clinically meaningful benefit and supporting potential labeling claims [33, 42]. As such, the method for identification of the MWPC of a cognitive PerfO developed and applied here can be extended to investigations of other cognitive PerfOs in NT1 and, more broadly, to cognitive PerfOs used in diseases or disorders for which cognitive function is a target of treatment.

In the current study, the PVT MWPC estimates were derived from changes anchored by multiple related PROs and ClinROs. While the use of multiple anchors and the triangulation method recommended by Trigg and Griffiths (2021) [41] increases the reliability of the estimates obtained, future studies seeking to replicate the MWPC results developed here could do this using additional analytic methods such as predictive modelling [45]. MWPC thresholds are specific to the patient population in which they were estimated. Thus, while the estimates in this study were generated from, and applied to, data from 2 independent studies, estimates from the current analysis are limited by the characteristics of the patient groups on which they are based. As such, the MWPC estimated here as a reduction in 2 lapses on the PVT may not be generalizable to all people with NT1. Future studies could use simulations based on a range of treatment effects as well as shifts in underlying population distributions to confirm the MWPC.

It is important to also note that there was variability in PVT change between the clinical trials and in observed change by anchor category. This variability was due in part to the smaller sample sizes for intermediate categories on some of the PROs and ClinROs (Table 3). To overcome this variability, the PVT MWPC was determined from change on all PROs/ClinROs and then triangulated against statistically based estimates of change beyond measurement error based on baseline within-subject variability. This broad approach provided a PVT MWPC estimate and range that was consistent across the various methods employed and which showed precision when applied in the unblinded responder analyses by study arms, with a high level of separation between treated and untreated groups in the frequency of clinically meaningful change as determined by the MWPC.

## Conclusion

In summary, these results indicate that oveporexton treatment produced clinically meaningful improvements in attention among adults with NT1. The MWPC threshold of −2 PVT lapses links the effects of oveporexton to objective and subjective aspects of cognitive symptoms, thereby providing a robust framework for interpreting cognitive benefits of orexin-targeted therapies. Future research should examine the durability of these effects, as well as their relationships with other cognition domains shown to be impaired in NT1 and assessed in clinical trials (e.g., memory, executive function). The approach used here also provides a strong methodological foundation for estimation of MWPC thresholds for changes in cognitive symptoms in related disorders, such as narcolepsy type 2 and idiopathic hypersomnia.

## Supplementary Information

Below is the link to the electronic supplementary material.

Supplementary Material 1

## Data Availability

The datasets generated during and/or analyzed during the current study are available from the corresponding author on reasonable request. Data will be shared only after deidentification, in compliance with applicable privacy laws, data protection regulation, and consent requirements.

## References

[1] Scammell TE, Narcolepsy. N Engl J Med. 2015;373(27):2654–62. 10.1056/NEJMra1500587. 26716917

[2] Barateau L, Pizza F, Plazzi G, et al. Narcolepsy. J Sleep Res. 2022;31(4):e13631. 10.1111/jsr.13631 35624073

[3] Cano CA, Harel BT, Scammell TE. Impaired cognition in narcolepsy: clinical and neurobiological perspectives. Sleep. 2024;47(9):zsae150. 10.1093/sleep/zsae150. PMC12477116 38943485

[4] Harel BT, Gattuso JJ, Latzman RD, et al. The nature and magnitude of cognitive impairment in narcolepsy type 1, narcolepsy type 2, and idiopathic hypersomnia: a meta-analysis. Sleep Adv. 2024;5(1):zpae043. 10.1093/sleepadvances/zpae043. PMC11258808 39036743

[5] Maski K, Steinhart E, Williams D, et al. Listening to the patient voice in narcolepsy: diagnostic delay, disease burden, and treatment efficacy. J Clin Sleep Med. 2017;13(3):419–25. 10.5664/jcsm.6494. PMC5337589 27923434

[6] Ortiz LE, Morse AM, Krahn L, et al. A survey of people living with narcolepsy in the USA: path to diagnosis, quality of life, and treatment landscape from the patient’s perspective. CNS Drugs. 2025;39(Suppl 1):23–36. 10.1007/s40263-024-01142-8. PMC11950095 40111736

[7] Wardle-Pinkston S, Slavish DC, Taylor DJ. Insomnia and cognitive performance: a systematic review and meta-analysis. Sleep Med Rev. 2019;48:101205. 10.1016/j.smrv.2019.07.008. 31522135

[8] Vaessen TJA, Mark RE, Overeem S, et al. Cognitive complaints in patients with suspected obstructive sleep apnea are associated with sleepiness, fatigue, and anxiety, not with final diagnosis or objective cognitive impairment. Clocks Sleep. 2025;7(1):12. 10.3390/clockssleep7010012. PMC11941010 40136849

[9] Bassetti CLA, Adamantidis A, Burdakov D, et al. Narcolepsy — clinical spectrum, aetiopathophysiology, diagnosis and treatment. Nat Rev Neurol. 2019;15(9):519–39. 10.1038/s41582-019-0226-9. 31324898

[10] American Academy of Sleep Medicine. International Classification of Sleep Disorders. 3rd ed. Darien, IL: American Academy of Sleep Medicine; 2014.

[11] Lammers GJ, Plazzi G, Mignot E, et al. Effects of oveporexton, an orexin receptor 2-selective agonist, on cognition in narcolepsy type 1: a secondary analysis of a randomized clinical trial. JAMA Neurol. 2026;83(2):145–52. 10.1001/jamaneurol.2025.4825. PMC12687147 41359331

[12] Mignot E, Arnulf I, Plazzi G, et al. The First Light: efficacy and safety of a multi-dose study of oveporexton (TAK-861), an oral orexin receptor 2 agonist, for the treatment of narcolepsy type 1. Sleep Med. 2026;138(Suppl):107565. 10.1016/j.sleep.2025.107565.

[13] Dauvilliers Y, Antczak J, Buntinx E, et al. The Radiant Light: efficacy and safety of oveporexton (TAK-861), an oral orexin receptor 2 agonist, for the treatment of narcolepsy type 1. Sleep Med. 2026;139(Suppl):107567. 10.1016/j.sleep.2025.107567.

[14] Harel B, Maruff P, Maski K, et al. 0812 Use of the Psychomotor Vigilance Test in clinical trials of CNS-active drugs: a systematic review and meta-analysis. Sleep. 2025;48(Suppl 1):A352. 10.1093/sleep/zsaf090.0812.

[15] Austin EE, Do V, Nullwala R, et al. Systematic review of the factors and the key indicators that identify doctors at risk of complaints, malpractice claims or impaired performance. BMJ Open. 2021;11(8):e050377. 10.1136/bmjopen-2021-050377. PMC8386219 34429317

[16] Rahman SA, Sullivan JP, Barger LK, et al. Extended work shifts and neurobehavioral performance in resident-physicians. Pediatrics. 2021;147(3):e2020009936. 10.1542/peds.2020-009936. PMC7919117 33619044

[17] St Hilaire MA, Anderson C, Anwar J, et al. Brief (< 4 hr) sleep episodes are insufficient for restoring performance in first-year resident physicians working overnight extended-duration work shifts. Sleep. 2019;42(5):zsz041. 10.1093/sleep/zsz041. PMC6519906 30794317

[18] Anderson C, Sullivan JP, Flynn-Evans EE, et al. Deterioration of neurobehavioral performance in resident physicians during repeated exposure to extended duration work shifts. Sleep. 2012;35(8):1137–46. 10.5665/sleep.2004. PMC3397817 22851809

[19] Wingelaar-Jagt YQ, Wingelaar TT, Riedel WJ, et al. Fatigue in aviation: safety risks, preventive strategies and pharmacological interventions. Front Physiol. 2021;12:712628. 10.3389/fphys.2021.712628. PMC8451537 34552504

[20] Behrens T, Burek K, Pallapies D, et al. Decreased psychomotor vigilance of female shift workers after working night shifts. PLoS One. 2019;14(7):e0219087. 10.1371/journal.pone.0219087 PMC6611661 31276523

[21] Rosa DE, Marot LP, de Mello MT, et al. Shift rotation, circadian misalignment and excessive body weight influence psychomotor performance: a prospective and observational study under real life conditions. Sci Rep. 2019;9(1):19333. 10.1038/s41598-019-55114-w. PMC6920148 31852906

[22] Huang Y, Hennig S, Fietze I, et al. The Psychomotor Vigilance Test compared to a divided attention steering simulation in patients with moderate or severe obstructive sleep apnea. Nat Sci Sleep. 2020;12:509–24. 10.2147/NSS.S256987. PMC7419631 32821184

[23] Mollicone D, Kan K, Mott C, et al. Predicting performance and safety based on driver fatigue. Accid Anal Prev. 2019;126:142–5. 10.1016/j.aap.2018.03.004. 29622267

[24] Alfonsi V, Palmizio R, Rubino A, et al. The association between school start time and sleep duration, sustained attention, and academic performance. Nat Sci Sleep. 2020;12:1161–72. 10.2147/NSS.S273875. PMC7735793 33328774

[25] Lerman S, Mollicone D, Coats S. Use of the Psychomotor Vigilance Test in fitness for work assessments. J Occup Environ Med. 2017;59(8):716–20. 10.1097/JOM.0000000000001065. 28692008

[26] Basner M, Rubinstein J. Fitness for duty: a 3-minute version of the Psychomotor Vigilance Test predicts fatigue-related declines in luggage-screening performance. J Occup Environ Med. 2011;53(10):1146–54. 10.1097/JOM.0b013e31822b8356. PMC3190077 21912278

[27] Basner M, Dinges DF, Mollicone D, et al. Mars 520-d mission simulation reveals protracted crew hypokinesis and alterations of sleep duration and timing. Proc Natl Acad Sci U S A. 2013;110(7):2635–40. 10.1073/pnas.1212646110. PMC3574912 23297197

[28] Basner M, Dinges DF, Mollicone DJ, et al. Psychological and behavioral changes during confinement in a 520-day simulated interplanetary mission to Mars. PLoS One. 2014;9(3):e93298. 10.1371/journal.pone.0093298 PMC3968121 24675720

[29] Basner M, Savitt A, Moore TM, et al. Development and validation of the Cognition Test Battery for spaceflight. Aerosp Med Hum Perform. 2015;86(11):942–52. 10.3357/AMHP.4343.2015. PMC4691281 26564759

[30] Lammers GJ, Maruff P, Banerjee T, et al. Characterization of the nature, severity, and subjective experience of cognitive symptoms associated with narcolepsy type 1. Sleep Med. 2025;138(Suppl):107494. 10.1016/j.sleep.2025.107494.

[31] Pizza F, Dauvilliers Y, del Rio Villegas R et al. Oveporexton (TAK-861) improves cognitive symptoms in patients with narcolepsy type 1: results from two randomized, placebo-controlled phase 3 trials. Sleep 2026;49(1):A329. 10.1093/sleep/zsag091.0737

[32] Revicki D, Hays RD, Cella D, et al. Recommended methods for determining responsiveness and minimally important differences for patient-reported outcomes. J Clin Epidemiol. 2008;61(2):102–9. 10.1016/j.jclinepi.2007.03.012. 18177782

[33] U.S. Department of Health and Human Services. Food and Drug Administration, Center for Drug Evaluation and Research (CDER), FDA draft guidance. Patient-focused drug development: incorporating clinical outcome assessments into endpoints for regulatory decision-making. Guidance for industry, Food and Drug Administration Staff, and other stakeholders. https://www.fda.gov/media/166830/download. Accessed 1 April 2023.

[34] Meunier J, Creel K, Loubert A, et al. Defining a clinically meaningful within-patient change threshold for the Cohen-Mansfield Agitation Inventory in Alzheimer’s dementia. Front Neurol. 2024;15:1379062. 10.3389/fneur.2024.1379062. PMC11301780 39108660

[35] Cohen J. Statistical Power Analysis for the Behavioral Sciences. 2nd ed. New York, NY: Routledge; 1988.

[36] Romero H, Savva Y, Doll H, et al. Establishing psychometric evidence of the novel patient-reported Functional Impacts of Narcolepsy Instrument (FINI) using data from observational and phase 2 clinical trials in narcolepsy. J Sleep Res. 2024;33(S1):517–8.

[37] Iverson GL, Lam RW. Rapid screening for perceived cognitive impairment in major depressive disorder. Ann Clin Psychiatry. 2013;25(2):135–40. 23638444

[38] Fayers PM, Hays RD. Don’t middle your MIDs: regression to the mean shrinks estimates of minimally important differences. Qual Life Res. 2014;23(1):1–4. 10.1007/s11136-013-0443-4. PMC3961825 23722635

[39] Hudgens S, Cappelleri JC, Coon C et al. PRO Consortium Webinar: Methods for determining clinically meaningful change. https://c-path.org/methods-for-determining-clinically-meaningful-change/. Accessed 1 April 2026.

[40] Froud R, Abel G. Using ROC curves to choose minimally important change thresholds when sensitivity and specificity are valued equally: the forgotten lesson of Pythagoras. Theoretical considerations and an example application of change in health status. PLoS One 2014;9(12):e114468. 10.1371/journal.pone.0114468 PMC4256421 25474472

[41] Trigg A, Griffiths P. Triangulation of multiple meaningful change thresholds for patient-reported outcome scores. Qual Life Res. 2021;30(10):2755–64. 10.1007/s11136-021-02957-4. 34319532

[42] European Medicines Agency. Reflection paper on patient experience data. https://www.ema.europa.eu/en/documents/scientific-guideline/reflection-paper-patient-experience-data_en.pdf. Accessed 2 April 2026.

[43] Takeda Pharmaceuticals. TK1075 PVT psychometric report v1.0. Takeda Clinical Outcomes Team.

[44] Villano I, Messina A, Valenzano A, et al. Basal forebrain cholinergic system and orexin neurons: effects on attention. Front Behav Neurosci. 2017;11:10. 10.3389/fnbeh.2017.00010. PMC5281635 28197081

[45] Terluin B, Eekhout I, Terwee CB, et al. Minimal important change (MIC) based on a predictive modeling approach was more precise than MIC based on ROC analysis. J Clin Epidemiol. 2015;68(12):1388–96. 10.1016/j.jclinepi.2015.03.015. 25913670

