# Supplementary material for: Defining a Clinically Meaningful Within-Person Change Threshold for the Psychomotor Vigilance Test in Narcolepsy Type 1
Source: Ther Innov Regul Sci. 2026 Aug 10;60(6):1861–73. doi: 10.1007/s43441-026-01025-8 (PMC13722789; doi:10.1007/s43441-026-01025-8)
Supplement: Supplementary file 1 [file 43441_2026_1025_MOESM1_ESM.docx]

**Supplementary Materials**

# Supplementary Methods

## Assessment of Cognitive Symptoms

*Psychomotor Vigilance Test (PVT)*

The PVT is a computerized 10-minute performance outcome measure that objectively quantifies sustained attention. During the task, participants respond to visual stimuli appearing at random intervals ranging from 2 to 10 seconds, and lapses of attention are defined as responses with reaction times > 500 ms [1]. To reduce the influence of skewness on the distribution of lapse counts, values were square root–transformed before statistical analysis and subsequently back-transformed for interpretability. The PVT was administered twice daily, once in the morning (9:00 AM) and once in the afternoon (3:00 PM). All assessments occurred in a quiet, controlled environment under the supervision of a trained test administrator. The mean of the 2 daily administrations was used as the endpoint.

*Functional Impacts of Narcolepsy Instrument – Cognitive Functioning (FINI-CF)*

The FINI is a 28-item patient-reported outcomes measure designed to assess the impact of narcolepsy symptoms on daily functioning across the following 6 domains: Tiredness, Cognitive Functioning (CF), Cataplexy, Social Activities, Everyday Activities, and Everyday Responsibilities [2]. The CF domain includes 5 items that evaluate patients’ perceived difficulty concentrating, maintaining mental sharpness, remembering things, staying focused, and thinking clearly over the past 7 days. Each item is rated on a 5-point Likert scale (0 = “never” to 4 = “always”), and domain scores are calculated by averaging item responses and linearly transforming the average to a 0–100 scale, with higher scores reflecting greater cognitive impairment. For this analysis, potential anchor measures were the individual Item 9 “Difficulty Concentrating” domain scores of the FINI and the FINI-CF total score. Change scores were calculated as the difference between baseline and post treatment (days 56 and 84) FINI Item 9 and FINI-CF scores, where negative values represent improvement.

*British Columbia Cognitive Complaints Inventory – Expanded (BC-CCI-E)*

The BC-CCI-E is an expanded version of the original 6-item British Columbia Cognitive Complaints Inventory [3], capturing the broader functional impact of cognitive symptoms. The core BC-CCI evaluates 6 cognitive domains (concentration, memory, word-finding, thought expression, problem-solving, and processing speed), each rated on a 4-point scale from 0 = “not at all” to 3 = “very much.” Total scores (0–18) quantify the severity of perceived cognitive difficulties, with higher scores indicating greater impairment. The total score can be categorized into the following 4 categories of cognitive complaint severity: 0–4 = broadly normal; 5–8 = mild cognitive complaints; 9–14 = moderate cognitive complaints; and 15–18 = severe cognitive complaints [3]. The expanded version (BC-CCI-E) includes 3 supplementary items capturing the following functional and quality-of-life consequences of cognitive complaints: difficulty with work/school performance, strain in personal relationships, and reduction in social or recreational engagement. Expanded items were not included in the BC-CCI-E total score calculation.

*Patient Global Impression of Severity (PGI-S) Narcolepsy*

The PGI-S Narcolepsy is a single-item patient-reported outcome measure that asks participants to report the severity of their overall narcolepsy symptoms over the previous 7 days on a 5-point response scale (1 = “none”; 2 = “mild”; 3 = “moderate”; 4 = “severe”; 5 = “very severe”).

*Patient Global Impression of Change (PGI-C) Narcolepsy*

The PGI-C Narcolepsy is a single-item patient-reported outcome measure that asks the participant to rate the change in their overall narcolepsy symptoms since starting the study medication on a 7-point scale (1 = “very much improved”; 2 = “much improved”; 3 = “minimally improved”; 4 = “no change”; 5 = “minimally worse”; 6 = “very much worse”; 7 = “very much worse”).

*Clinician Global Impression of Severity (CGI-S) Narcolepsy*

The CGI-S Narcolepsy is a single-item clinician-rated measure to assess the clinical impression of the participant’s severity of overall narcolepsy symptoms at the time of the assessment using a 7-point response scale (1 = “normal, not at all ill”; 2 = “borderline ill”; 3 = “mildly ill”; 4 = “moderately ill”; 5 = “markedly ill”; 6 = “severely ill”; 7 = “among the most extremely ill”).

*Clinician Global Impression of Change (CGI-C) Narcolepsy*

The CGI-C Narcolepsy is a single-item clinician-rated measure to assess the clinical impression of change in the participant’s overall narcolepsy symptoms compared with baseline using a 7-point Likert-type scale (1 = “very much improved”; 2 = “much improved”; 3 = “minimally improved”; 4 = “no change”; 5 = “minimally worse”; 6 = “very much worse”; 7 = “very much worse”).

# References

1.	Cohen J. Statistical Power Analysis for the Behavioral Sciences. 2nd ed. New York, NY: Routledge; 1988.

2.	Romero H, Savva Y, Doll H, et al. Establishing psychometric evidence of the novel patient-reported Functional Impacts of Narcolepsy Instrument (FINI) using data from observational and phase 2 clinical trials in narcolepsy. J Sleep Res. 2024;33(S1):517–8.

3.	Iverson GL, Lam RW. Rapid screening for perceived cognitive impairment in major depressive disorder. Ann Clin Psychiatry. 2013;25(2):135–40.

**Appendix Table 1.** Correlations* between change in square root PVT lapses and change in potential anchor measures from baseline to days 56 and 84.

| **Anchor measure** | Day 56 | | Day 84 | |
| --- | --- | --- | --- | --- |
|  | The First Light  (n = 159) | The Radiant Light  (n = 103) | The First Light  (n = 156) | The Radiant Light  (n = 100) |
| **Concept-specific anchors** | | | | |
| **FINI – Cognitive Functioning domain** | **0.372** | **0.379** | **0.461** | 0.286 |
| **FINI-Difficulty Concentrating** | **0.330** | 0.**327** | **0.390** | 0.233 |
| **BC-CCI-E total score** | **0.400** | **0.411** | **0.322** | **0.408** |
| BC-CCI-E Item 2: Poor Concentration | **0.329** | **0.366** | **0.309** | **0.392** |
| BC-CCI-E Item 7: Difficult for Me to Do My Job | **0.322** | 0.269 | 0.284 | 0.208 |
| BC-CCI-E Item 8: Difficult to Have a Good Relationship | **0.346** | 0.200 | **0.312** | 0.150 |
| BC-CCI-E Item 9: Difficult to Enjoy Social Activity | **0.335** | 0.230 | **0.300** | 0.209 |
| Global anchors | | | | |
| PGI-S | **0.310** | **0.403** | 0.263 | **0.350** |
| PGI-C | **0.307** | **0.453** | 0.262 | **0.402** |
| CGI-S | **0.344** | **0.440** | 0.221 | **0.337** |
| CGI-C | **0.445** | **0.469** | **0.312** | **0.395** |

BC-CCI-E, British Columbia Cognitive Complaints Inventory – Extended; FINI, Functional Impacts of Narcolepsy Instrument; CGI-C, Clinician Global Impression of Change; CGI-S, Clinician Global Impression of Severity; PGI-C, Patient Global Impression of Change; PGI-S, Patient Global Impression of Severity; PVT, Psychomotor Vigilance Test.

*Moderate correlations (≥ 0.30) are presented in **bold**.

**Appendix Table 2**: Receiver Operating Characteristic (ROC) Best Cut-Points (BCPs) for a 1 +, 2 +, or 3 + Category Change in each Concept-Specific Anchor at Day 84 for The First Light (N = 166) and The Radiant Light (N = 105)

| **SQRT PVT BCP** | **FINI Item 9** | | | | **FINI CF** | | | | **BC-CCI-I Item 2** | | | | **BC-CCI-E Item 7** | | | | **BC-CCI-E Item 8** | | | | **BC-CCI-E Item 9** | | | | **BC-CCI-E Total Score** | | | | **Specific**  **Mean** |
| --- | --- | --- | --- | --- | --- | --- | --- | --- | --- | --- | --- | --- | --- | --- | --- | --- | --- | --- | --- | --- | --- | --- | --- | --- | --- | --- | --- | --- | --- |
|  | **d(0,1)** | **S-S** | **YI** | **Mean** | **d(0,1)** | **S-S** | **YI** | **Mean** | **d(0,1)** | **S-S** | **YI** | **Mean** | **d(0,1)** | **S-S** | **YI** | **Mean** | **d(0,1)** | **S-S** | **YI** | **Mean** | **d(0,1)** | **S-S** | **YI** | **Mean** | **d(0,1)** | **S-S** | **YI** | **Mean** |  |
| **The First Light** |  |  |  |  |  |  |  |  |  |  |  |  |  |  |  |  |  |  |  |  |  |  |  |  |  |  |  |  |  |
| **Day 84** |  |  |  |  |  |  |  |  |  |  |  |  |  |  |  |  |  |  |  |  |  |  |  |  |  |  |  |  |  |
| 3 + change | −1.41 | −1.18 | −0.39 | −0.99 | −1.00 | −1.00 | −0.39 | −0.80 | −1.42 | −1.42 | −2.65 | −1.83 | −1.01 | −1.41 | −0.87 | −1.10 | −2.40 | −2.45 | −2.40 | −2.42 | −2.56 | −1.74 | −2.56 | −2.29 | −2.65 | −2.40 | −2.65 | −2.57 | **−1.71** |
| 2 + change | −1.00 | −1.00 | −0.39 | −0.80 | −0.39 | −0.87 | −0.39 | −0.55 | −1.01 | −1.32 | −0.19 | −0.84 | −1.42 | −1.22 | −1.42 | −1.35 | −1.41 | −1.42 | −0.42 | −1.08 | −1.42 | −1.18 | −0.37 | −0.99 | −1.22 | −1.32 | −0.99 | −1.18 | **−0.97** |
| 1 + change | −0.39 | −0.63 | −0.39 | −0.47 | −0.39 | −0.71 | −0.39 | −0.50 | −0.99 | −0.81 | −0.99 | −0.93 | −1.42 | −0.97 | −1.41 | −1.27 | −0.87 | −1.00 | −0.87 | −0.91 | −0.67 | −0.81 | 0.29 | −0.40 | −0.99 | −0.97 | −0.29 | −0.75 | **−0.75** |
| **The Radiant light** |  |  |  |  |  |  |  |  |  |  |  |  |  |  |  |  |  |  |  |  |  |  |  |  |  |  |  |  |  |
| **Day 84** |  |  |  |  |  |  |  |  |  |  |  |  |  |  |  |  |  |  |  |  |  |  |  |  |  |  |  |  |  |
| 3 + change | −1.41 | −1.18 | −0.39 | −0.99 | −0.36 | −0.36 | 0.66 | −0.02 | −0.36 | −0.41 | 0.58 | −0.06 |  |  |  |  |  |  |  |  |  |  |  |  | −0.41 | −0.52 | −0.41 | −0.45 | **−0.41** |
| 2 + change | −1.00 | −1.00 | −0.39 | −0.80 | −0.19 | −0.19 | −1.29 | −0.56 | −0.36 | −0.52 | −0.17 | −0.35 |  |  |  |  |  |  |  |  |  |  |  |  | −0.36 | −0.52 | −0.36 | −0.41 | **−0.47** |
| 1 + change | −0.39 | −0.63 | −0.39 | −0.47 | −0.19 | −0.18 | −0.87 | −0.41 | −0.14 | −0.18 | −0.06 | −0.13 |  |  |  |  |  |  |  |  |  |  |  |  | −0.17 | −0.36 | −0.17 | −0.23 | **−0.29** |

BC-CCI-E = British Columbia Cognitive Complaints Inventory – Extended; BCP = best cut point; d(0,1) = distance from the 0,1 ROC space; FINI = Functional Impacts of Narcolepsy Instrument; S-S = sensitivity minus specificity; SQRT = square root; PVT = Psychomotor Vigilance Test; YI = Youden’s Index

**Appendix Table 3:** Receiver Operating Characteristic (ROC) Best Cut-Points (BCPs) for a 1 +, 2 +, or 3 + Category Change in each Global Anchor at Day 84 for The First Light (*N* = 166) and The Radiant Light (*N* = 105)

| **SQRT PVT BCP** | **PGI-S** | | | | **PGI-C** | | | | **GCI-S** | | | | **CGI-C** | | | | **Global**  **Mean** | **Overall Mean** |
| --- | --- | --- | --- | --- | --- | --- | --- | --- | --- | --- | --- | --- | --- | --- | --- | --- | --- | --- |
|  | **d(0,1)** | **S-S** | **YI** | **Mean** | **d(0,1)** | **S-S** | **YI** | **Mean** | **d(0,1)** | **S-S** | **YI** | **Mean** | **d(0,1)** | **S-S** | **YI** | **Mean** |  |  |
| **The First Light** |  |  |  |  |  |  |  |  |  |  |  |  |  |  |  |  |  |  |
| **Day 84** |  |  |  |  |  |  |  |  |  |  |  |  |  |  |  |  |  |  |
| 3 + change | −0.76 | −1.08 | −0.76 | −0.87 | −0.39 | −1.06 | −0.37 | −0.61 | −0.81 | −1.00 | −0.81 | −0.87 | −0.65 | −1.13 | −0.65 | −0.81 | −0.79 | −1.38 |
| 2 + change | −0.65 | −1.01 | −0.42 | −0.69 | −0.65 | −0.71 | 0.79 | −0.19 | −0.65 | −0.71 | −0.65 | −0.67 | −0.65 | −0.80 | −0.65 | −0.70 | −0.56 | −0.82 |
| 1 + change | −1.01 | −0.65 | −1.01 | −0.89 | −0.65 | −0.50 | −0.65 | −0.60 | −0.60 | −0.42 | 0 | −0.34 | −0.15 | −0.39 | −0.15 | −0.23 | −0.52 | −0.66 |
| **The Radiant Light** |  |  |  |  |  |  |  |  |  |  |  |  |  |  |  |  |  |  |
| **Day 84** |  |  |  |  |  |  |  |  |  |  |  |  |  |  |  |  |  |  |
| 3 + change | −1.29 | −0.41 | −1.29 | −1.00 | −0.87 | −0.41 | −0.87 | −0.72 | −0.17 | −0.41 | −0.17 | −0.25 | −0.36 | −0.59 | −0.36 | −0.44 | −0.60 | −0.51 |
| 2 + change | −0.36 | −0.36 | −0.36 | −0.36 | −0.19 | −0.08 | −0.19 | −0.15 | −0.19 | −0.17 | −0.19 | −0.18 | −0.19 | −0.13 | −0.19 | −0.17 | −0.22 | −0.35 |
| 1 + change | 0.45 | −0.05 | 0.45 | 0.28 | −0.19 | −0.05 | −0.19 | −0.14 | −0.06 | −0.05 | −0.19 | −0.10 | −0.19 | −0.08 | −0.19 | −0.15 | −0.03 | −0.16 |

BCP = best cut point; CGI-C = Clinician Global Impression of Change; CGI-S = Clinician Global Impression of Severity; d(0,1) = distance from the 0,1 ROC space; PGI-C = Patient Global Impression of Change; PGI-S = Patient Global Impression of Severity; S-S = sensitivity minus specificity; SQRT = square root; PVT = Psychomotor Vigilance Test; YI = Youden’s Index

**Appendix Fig. 1** eCDF curves of the change in square root PVT lapses from baseline to day 84 for the PGI-S and PGI-C anchors in The First Light (*N* = 166) and The Radiant Light (*N* = 105).

eCDF, empirical cumulative distribution function; PGI-C, Patient Global Impression of Change; PGI-S, Patient Global Impression of Severity; PVT, Psychomotor Vigilance Test.

**Appendix Fig. S2** eCDF curves of the change in square root PVT lapses from baseline to day 84 for the CGI-S and CGI-C anchors in The First Light (*N* = 166) and The Radiant Light (*N* = 105).

CGI-C, Clinician Global Impression of Change; CGI-S, Clinician Global Impression of Severity; eCDF, empirical cumulative distribution function; PVT, Psychomotor Vigilance Test.

**Appendix Figure 3.** PDF curves of the change in square root PVT lapses from baseline to day 84 for the PGI-S and PGI-C anchors in The First Light (*N* = 166) and The Radiant Light (*N* = 105).

PDF, probability density function; PGI-C, Patient Global Impression of Change; PGI-S, Patient Global Impression of Severity; PVT, Psychomotor Vigilance Test.

**Appendix Figure 4.** PDF curves of the change in square root PVT lapses from baseline to day 84 for the CGI-S and CGI-C anchors in The First Light (*N* = 166) and The Radiant Light (*N* = 105).

CGI-C, Clinician Global Impression of Change; CGI-S, Clinician Global Impression of Severity; PDF, probability density function; PVT, Psychomotor Vigilance Test.

**Appendix Figure 5:** Illustrative Receiver Operating Characteristic (ROC) Curves for Concept-Specific Anchors at Day 84 for The First Light (*N* = 166) and The Radiant Light (*N* = 105)

| **The First light** | **The Radiant Light** |
| --- | --- |
| **BC-CCI-E Total Score** | |
|  |  |
| **BC-CCI-E Item 2 Poor Concentration** | |
|  |  |

| **FINI Cognitive Functioning** | | |
| --- | --- | --- |
|  |  | |
| **FINI Item 9 Difficulty Concentrating** | | |
|  | |  |

BC-CCI-E = British Columbia Cognitive Complaints Inventory – Extended; FINI = Functional Impacts of Narcolepsy Instrument.

**Appendix Figure 6:** Illustrative Receiver Operating Characteristic (ROC) Curves for Global Anchors at Day 84 for Studies PVT-S3001 (*N* = 166) and PVT-S3002 (*N* = 105)

| **The First light** | **The Radiant Light** |
| --- | --- |
| **PGI-S** | |
|  |  |
| **PGI-C** | |
|  |  |

| **CGI-S** | |
| --- | --- |
|  |  |
| **CGI-C** | |
|  |  |

CGI-C = Clinician Global Impression of Change, CGI-S = Clinician Global Impression of Severity; PGI-C = Patient Global Impression of Change; PGI-S = Patient Global Impression of Severity.
